# Changes in neurotransmitter levels and expression of immediate early genes in brain of mice infected with *Neospora caninum*

**DOI:** 10.1038/srep23052

**Published:** 2016-03-14

**Authors:** Fumiaki Ihara, Maki Nishimura, Yoshikage Muroi, Hidefumi Furuoka, Naoaki Yokoyama, Yoshifumi Nishikawa

**Affiliations:** 1 National Research Center for Protozoan Diseases, Obihiro University of Agriculture and Veterinary Medicine, Inada-cho, Obihiro, Hokkaido 080-8555, Japan; 2 Department of Basic Veterinary Medicine, Obihiro University of Agriculture and Veterinary Medicine, Inada-cho, Obihiro, Hokkaido 080-8555, Japan

## Abstract

*Neospora caninum* is an obligate intracellular parasite that causes neurological disorders in dogs and cattle. The majority of host animals are asymptomatic at the chronic stage of infection. However, it remains unclear whether cerebral function is normal in asymptomatic animals. In this study, mice were infected with *N. caninum* (strain Nc-1) and their brains were examined to understand changes in cerebral function at the chronic stage of infection. Mice infected with *N. caninum* showed impaired locomotor activity, but no differences in clinical symptoms were observed. In the brains of infected mice, parasites were distributed throughout the brain and histological lesions were observed everywhere except for the cerebellum. Expression levels of proinflammatory cytokines, interferon-gamma and tumour necrosis factor-alpha, were highly upregulated in several brain regions of infected mice. Additionally, the level of neurotransmitters glutamate, glycine, gamma-aminobutyric acid, dopamine and 5-hydroxytryptamine, were altered in infected mice compared with those of uninfected mice. Interestingly, the expression levels of immediately early genes, c-Fos and Arc, in the brain of infected mice were lower than those of in uninfected mice. Our findings may provide insight into neurological disorders associated with *N. caninum* infection.

*Neospora caninum* is an intracellular parasite that naturally infects dogs and cattle^1^. *N. caninum* is closely related to *Toxoplasma gondii*, and was reported for the first time in 1988^2^. There is no evidence of *N. caninum* infectivity in humans, but serological evidence suggests that humans can be exposed to *N. caninum*^3^. *N. caninum* causes a variety of neurological symptoms such as pelvic limb paralysis and rigid hyperextension, which are particular to, and often observed in dogs^4^,^5^,^6^. In cattle, *N. caninum* is one of the main causes of abortion, and calves vertically infected with the parasites show neurological disorders^1^.

Activated microglia produce pro-inflammatory cytokines, nitric oxide (NO) and excitatory amino acids^7^, and thus play a central role in the innate immune response in the central nervous system (CNS). These microglial responses are also strongly related to neuronal disorders and neurodegeneration. Neuroinflammatory activation can lead to altered levels of neurotransmitters such as glutamate (Glu), dopamine (DA), 5-hydroxytryptamine (5-HT). Furthermore, expression of immediately early genes (IEG) is regulated by neuronal activity. c-Fos is a regulatory transcription factor which can indirectly influence cell function depending on the genes that they regulate^8^,^9^,^10^. Expression of activity-dependent cytoskeletal protein (Arc) is induced in response to synaptic activity and implicated in synaptic plasticity^11^.

Information about pathogenicity and immune response of neosporosis has been derived mainly from rodent models^12^. Mice infected with *N. caninum* develop various kinds of nervous symptoms such as wryneck, circular movement, and paralytic gait^13^,^14^,^15^. Thus, mouse models seem to be suitable for analyses of brain pathology during *N. caninum* infection. However, few studies have been done to investigate the effects of *N. caninum* infection on neuronal disorder and levels of neurotransmitters. In our previous study, we investigated gene expression profiles and histopathological changes in the brain of BALB/c mice infected with *N. caninum*. Our findings indicated that *N. caninum* infection stimulated the immune response in the brain^16^, because BALB/c are susceptible to *N. caninum* and exhibit encephalitis caused by the infection^15^. Although natural hosts chronically infected with *N. caninum* are usually asymptomatic, the asymptomatic to symptomatic mechanisms of neosporosis onset is still unknown. C57BL/6 male mice are a suitable model for the asymptomatic condition because this strain is relatively resistant to intraperitoneal infection of *N. caninum* (strain Nc-1). Thus, to understand the onset mechanism of neosporosis, we examined sickness behaviour, parasite distribution, histopathological lesions, levels of neurotransmitters (monoamines and amino acids), and expression of IEGs using asymptomatic C57BL/6 male mice during subacute infection with *N. caninum*. Our findings provide insight into neosporosis associated with brain dysfunction via inflammation, dysregulation of neurotransmitters and downregulation of IEGs.

## Results

### Effect of *N. caninum* infection on mice behaviour

**Figure 1:**
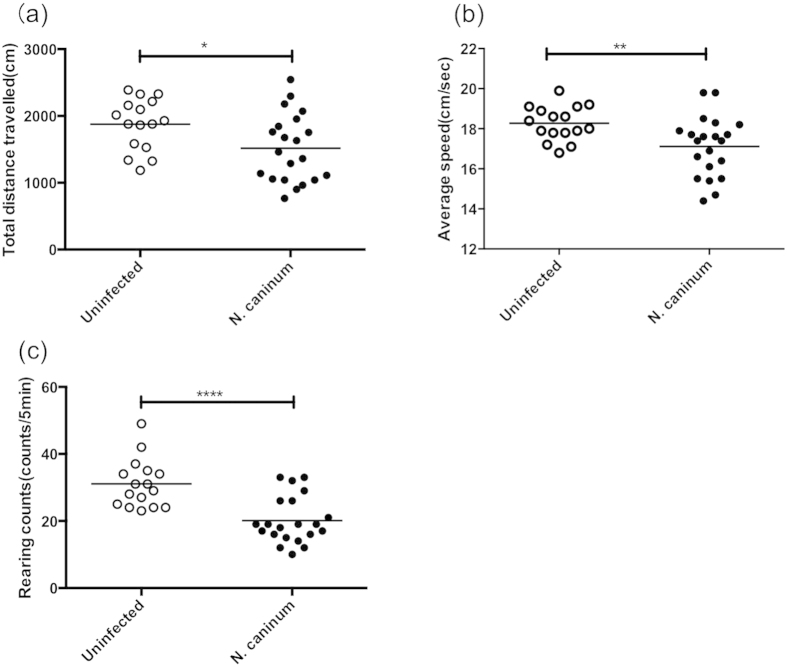
Effects of *N. caninum* infection on behaviour of mice. Changes in the locomotor activity and exploration parameters at 30 days post infection (dpi): (**a**) total distance travelled (cm), (**b**) average speed (cm/sec) and (**c**) rearing counts (counts/5 min). Data were summarised from two independent experiments. To reserve the number of mice required in *N. caninum*-infected group, each experiment used uninfected, n = 8; *N. caninum*-infected mice, n = 12. Because one mouse died at 28 dpi in *N. caninum*-infected, the total numbers of uninfected mice were n = 16; *N. caninum*-infected mice, n = 23. Significant differences were determined by unpaired t tests (*p < 0.05, **p < 0.01, ****p < 0.0001).

In our experimental model using C57BL/6 male mice, typical acute stage symptoms and neurological symptoms were not observed in the infected animals during the experimental period. However, infected mice showed differences in locomotor activity on the open field test (Fig. 1). Total distance travelled, average speed, and rearing counts were significantly decreased in mice infected with *N. caninum*. This result suggests *N. caninum* infection may affect the brain function of asymptomatic mice.

### Histopathological lesions and parasite distribution in distinct brain regions

**Figure 2:**
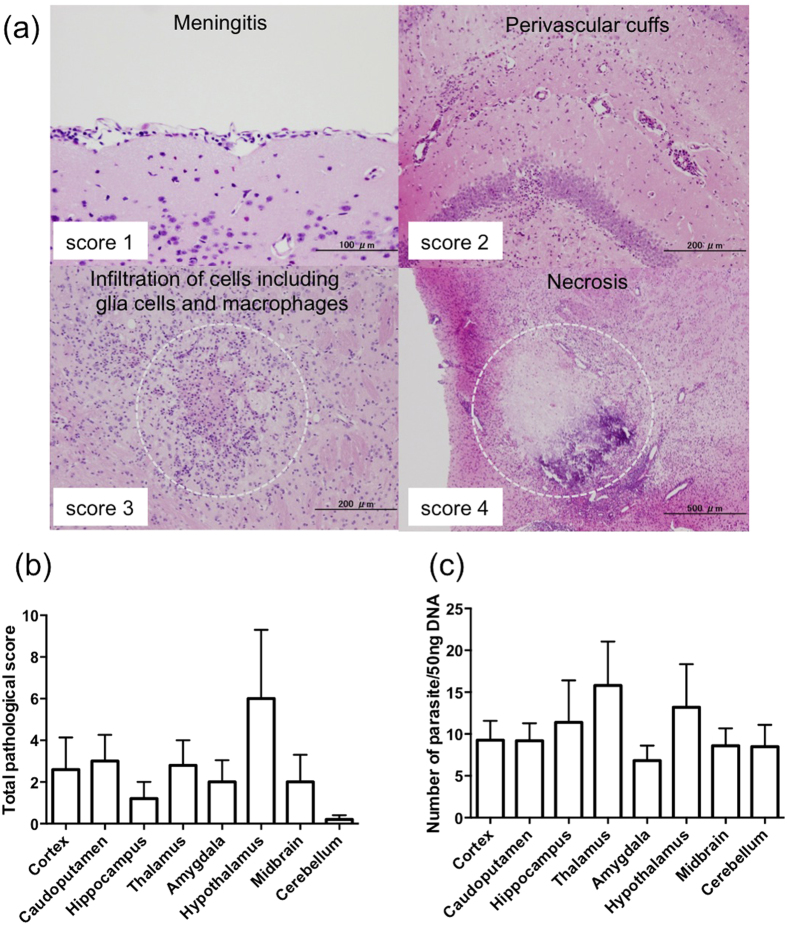
Histopathological lesions and parasite load in different brain regions of *N. caninum*-infected mice. (**a**) Representative examples of histopathological lesions from brains of *N. caninum*-infected mice. score 1: slight meningitis, score 2: mild perivascular cuffs, score 3: moderate inflammatory cell infiltration, score 4: extensive necrosis with rarefaction. (**b**) Total pathological score for each brain region. Brain samples were collected at 45 days post infection (dpi). Histopathological lesions were scored as above. Minimum value = 0 and maximum value = 16. *N. caninum*-infected mice, *n* = 5 from one experiment. (**c**) Parasite number per 50 ng tissue DNA. Brain samples were collected at 54 dpi. *N. caninum*-infected mice, *n* = 12 from one experiment. Significant differences were analysed by one-way ANOVA with Tukey’s post hoc test, but there were no significant differences.

We analysed eight regions of the brain histopathologically (Fig. 2). Histopathological lesions, including perivascular cuff, mononuclear cellular meningitis, glial cell activation, and focal necrosis were observed in the brains of infected mice. Although there were no significant differences between brain regions, the pathological score tended to be lower in the cerebellum compared with other regions (Fig. 2(b)). Next, parasite load in distinct brain regions was examined by quantitative PCR (Fig. 2(c)). *N. caninum* was observed in all brain regions examined, but there were no significant differences among the brain regions. This result indicates that *N. caninum* infection may induce wide-ranging histopathological lesions in the brains of asymptomatic mice.

### Expression levels of interferon-gamma (IFN-γ) and tumour necrosis factor-alpha (TNF-α) in brains of *N. caninum*-infected mice

**Figure 3:**
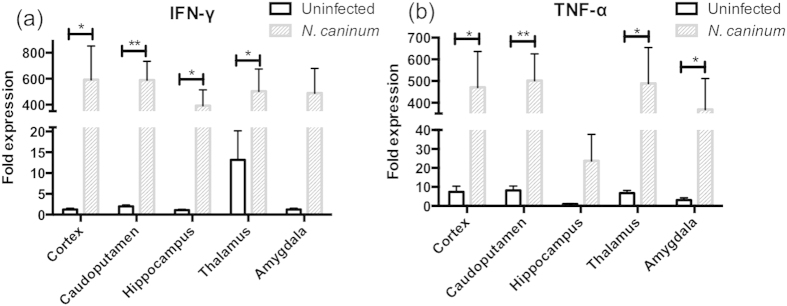
Expression levels of IFN-γ and TNF-α in the brain of *N. caninum*-infected mice. Brain samples of uninfected and *N. caninum*-infected mice were collected at 40 days post infection (dpi). Fold expression was determined by real-time PCR, and calculated relative to expression levels of the IFN-γ and TNF-α in the hippocampus of uninfected mice. Significant differences in each brain region between uninfected and *N. caninum*-infected group were determined by unpaired t tests (*p < 0.05, **p < 0.01). Significant differences among brain regions were analysed by one-way ANOVA with Tukey’s post hoc test, but there were no significant differences. Data represent mean ± SEM. Control, *n* = 6; *N. caninum*-infected mice, *n* = 6. Data are representative of two independent experiments.

We examined the expression levels of IFN-γ and TNF-α in five brain regions involved in neuroinflammation and CNS disorders mediated via activated microglia. Expression of IFN-γ was significantly upregulated in the cortex, hippocampus, striatum, and thalamus of *N. caninum*-infected mice compared with uninfected mice (Fig. 3(a)). Additionally, the expression level of TNF-α was significantly upregulated in the cortex, amygdala, striatum, and thalamus of the infected mice (Fig. 3(b)). However, there was no significant difference in the level of IFN-γ and TNF-α expression amongst brain regions. Although the expression level of TNF-α in hippocampus was low compared with the other regions, the mean TNF-α expression level in the hippocampus of infected mice was over 30 fold compared with that of uninfected animals. Increased expression of IFN-γ and TNF-α may be associated with histopathological lesions in brains of infected mice.

### Neurotransmitter levels in distinct brain regions of *N. caninum*-infected mice

**Figure 4:**
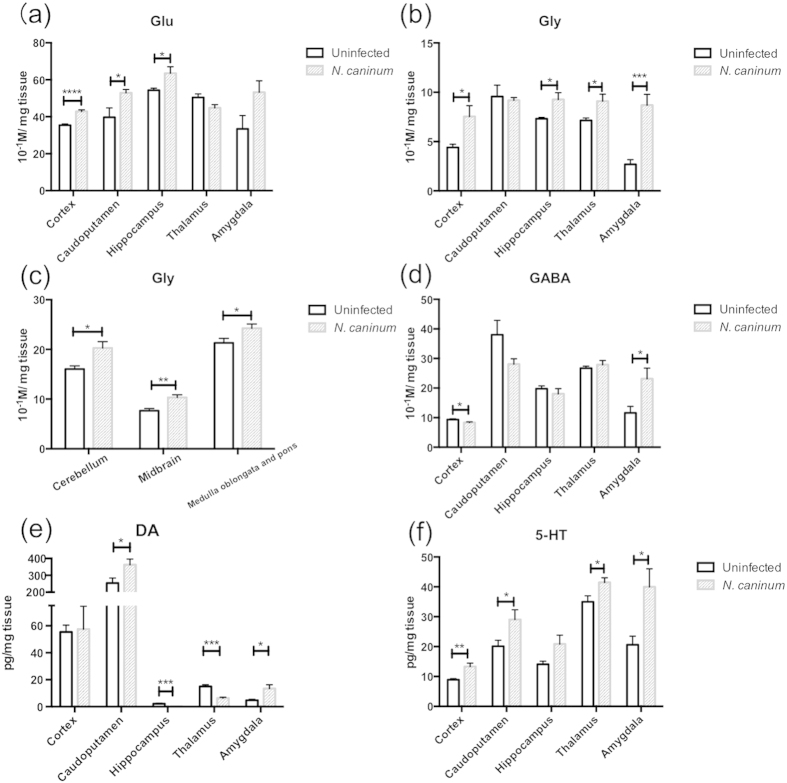
HPLC analysis of amino acids and monoamines. (**a**,**b**,**d**–**f**) Brain samples were collected at 40 days post infection and analysed by HPLC for glutamate (Glu), glycine (Gly), gamma-aminobutyric acid (GABA), dopamine (DA), 5-hydroxytryptamine (5-HT). Data represent mean ± SEM. Control, *n* = 6; *N. caninum*-infected mice, *n* = 6. Data are representative of two independent experiments. (**c)** Brain samples were collected at 52 days post infection and analysed by HPLC for Gly in hindbrain areas. Data represent mean ± SEM. Control, *n* = 8; *N. caninum*-infected mice, *n* = 12. Significant differences in each brain region between uninfected and *N. caninum*-infected group were determined using unpaired *t* tests (*p < 0.05, **p < 0.01, ***p < 0.001, ****p < 0.0001).

Next, we measured neurotransmitters in the brain. Levels of several neurotransmitters were altered in distinct brain regions of infected mice compared with uninfected mice (Fig. 4). Glu levels in the cortex, caudoputamen and hippocampus of *N. caninum* infected mice were significantly higher than those of uninfected animals (Fig. 4(a)). Glycine (Gly) levels in the cortex, hippocampus, thalamus and amygdala of infected mice were higher than those of uninfected animals (Fig. 4(b)). Since Gly is known to be an inhibitory neurotransmitter in the hindbrain, the levels of Gly in the medulla oblongata and pons, midbrain, and cerebellum were also analysed (Fig. 4(c)). Gly levels of infected mice were increased in these regions compared with uninfected animals. These results suggested that excess Gly neurotransmission in the brain occurred because of *N. caninum* infection. In comparison to uninfected mice, higher levels of gamma-aminobutyric acid (GABA) in the amygdala and lower levels in the cortex were observed in infected mice (Fig. 4(d)). In the case of the monoamine neurotransmitter DA, levels in the caudoputamen and amygdala were higher than those of uninfected animals. However, lower levels of DA in hippocampus and thalamus were seen in infected mice (Fig. 4(e)). In addition, higher 5-HT levels in the cortex, caudoputamen, amygdala and thalamus were observed in infected mice (Fig. 4(f)). Together, *N. caninum* infection may trigger a difference in neurotransmitter levels in several brain regions compared with uninfected mice.

### Expression levels of c-Fos and Arc in distinct brain regions of *N. caninum*-infected mice

**Figure 5:**
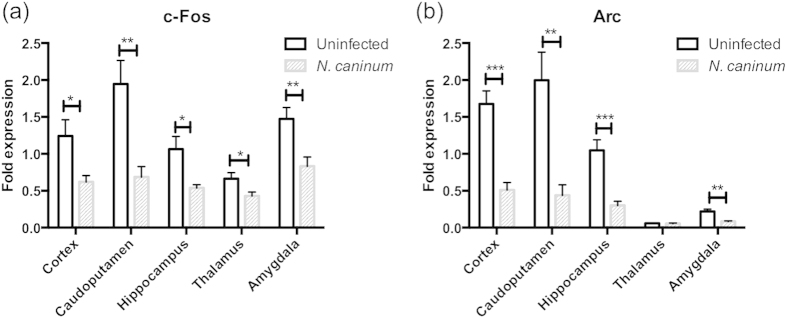
Expression levels of c-Fos and Arc in the brain of *N. caninum*-infected mice. Brain samples of uninfected and *N. caninum*-infected mice were collected at 40 days post infection. Fold expression was determined by real-time PCR, and calculated relative to expression levels of c-Fos and Arc in the hippocampus of uninfected mice. Significant differences in each brain region between uninfected and *N. caninum*-infected group were determined by unpaired t tests (*p < 0.05, **p < 0.01, ***p < 0.001). Data represent mean ± SEM. Control, *n* = 6; *N. caninum*-infected mice, *n* = 6. Data are representative of two independent experiments.

Finally, we examined expression levels of c-Fos and Arc because they have been used as markers for neuronal activation. Expression of c-Fos decreased in all regions of *N. caninum*-infected mice (Fig. 5(a)). Arc expression decreased in the cortex, caudoputamen, hippocampus and amygdala, but not in the thalamus of *N. caninum*-infected mice (Fig. 5(b)). These data indicate that *N. caninum* infection reduces expression of IEGs in the brain.

## Discussion

Generally, *N. caninum* infection in dogs causes encephalomyelitis, polyradiculoneuritis, pelvic limb paralysis, rigid hyperextension, and muscle atrophy^4^,^5^,^6^. *N. caninum* infection not only causes similar neurological signs in cattle but is also associated with abortions in cows^4^. Moreover, *N. caninum*-infected rodents develop various kinds of neurological signs such as wryneck, circular movement, and paralytic gait^13^,^14^,^15^. However, the mechanism of onset of neosporosis is largely unknown. Because neurological signs appear asymptomatic and then become symptomatic, it is important to study the brain of *N. caninum*-infected asymptomatic animals. In this study, *N. caninum*-infected mice showed low activity and exploratory behaviour in a novel environment, 30 days post infection (dpi). To our knowledge, our study is the first to report the behavioral change of mice infected with *N. caninum*. The behavioral change of rodents infected with *T. gondii*, which is closely related to *N. caninum*, has been well studied in the past decade. The effects of *T. gondii* infection on rodent behavior vary with experimental design. Time post infection may affect the activity level of mice^17^. For example, hypoactivity of infected animals was found within 2 to 3 months post infection^18^. In contrast, hyperactivity of infected animals was observed at a later clinical stage (3–7 months post infection)^19^,^20^,^21^,^22^. In addition, another study did not find any significant difference in activity at 3 months post infection^23^,^24^. Consequently, hypoactivity of mice infected with *T. gondii* were observed at an acute to sub-acute stage. It has been suggested that hypoactivity of infected mice was due to acute illness. Hyperactivity of infected mice was thought to be a result of histopathological damage associated with an immune response and development of cysts in the brain^23^. In this study we carried out open field tests at 4 weeks post infection and showed mild decrease of locomotor activity in mice infected with *N. caninum*. Although *N. caninum* infected mice showed no typical clinical symptoms and loss of body weight, they expressed high levels of inflammatory cytokines and histopathological damage in the brain. Thus, our results suggested that at 4 weeks post infection, *N. caninum*-infected mice had less activity levels because of sustained inflammatory response and histological damage of the brain.

Several studies have indicated that the brain stem and cerebellum show high sensitivity to *N. caninum* infection^25^,^26^,^27^,^28^. However, there was no association between parasite density and the severity of the lesion in those regions^16^. In the present study using asymptomatic mice, both tissue damage and parasite load did not show tropism. These results suggested that tissue damage associated with *N. caninum* infection had no region specificity in the brain during chronic stage. Previously, we found that the frontal lobe and medulla oblongata were mainly affected in symptomatic BALB/c mice infected with *N. caninum*, and some mice showed severe histopathological lesions in the cerebellum^16^. Therefore, lesion formation in the medulla oblongata and the cerebellum may be characteristic of neosporosis in mice. In the present study using asymptomatic C57BL/6 mice, lesion formation in the cerebellum was low compared with other regions, indicting the importance of histopathological lesions in the cerebellum for onset of neosporosis.

Our previous study showed that activation of microglia and increased expression level of inducible nitric oxide synthase were observed in the brains of mice infected with *N. caninum*^16^. In this study, expression of IFN-γ and TNF-α were highly upregulated in the brain of *N. caninum*-infected mice, suggesting neuroinflammation and CNS disorder via inflammation by immune cells. Generally, IFN-γ controls *N. caninum* proliferation in macrophage cultures^29^,^30^,^31^. Treatment of microglia and astrocytes with IFN-γ and TNF-α shows inhibition of parasite growth^32^. Moreover, IFN-γ and TNF-α inhibit *N. caninum* growth in a bovine cerebellar cell culture^33^. These responses induce neuronal disorder and neuronal cell death while they can eliminate parasites in tissues^29^,^32^,^33^,^34^. IFN-γ and TNF-α produced by immune cells disrupt mitochondrial ATP production and cause Glu excitotoxicity^35^,^36^. Excess Glu release induces neuronal cell death via N-methyl d-aspartate receptor^35^,^36^. Together, our results indicate chronic inflammation in the brain of asymptomatic C57BL/6 mice infected with *N. caninum*.

Pathogen infection into the brain can alter neurotransmission and cause dysfunction of this organ^37^. Some studies have suggested that *T. gondii* can alter DA metabolism and host behavior^38^,^39^. Stibbs showed that DA levels were 14% higher in mice with chronic infections than controls^40^. Gatokowska *et al*. suggested that an increase of 5-HT and the DA system might be responsible for low activity and exploratory behaviour of mice^41^. These results suggest differences in several neurotransmitter levels of mice infected with *T. gondii*. In this study, we examined changes in neurotransmitter levels in brain tissue following *N. caninum* infection for the first time. Our results showed that *N. caninum* infection induced marked change of excitatory and inhibitory neurotransmitter levels in wide brain areas, and these responses may induce sickness behaviour in infected mice. The amount of Glu was increased in the cortex, caudoputamen, and hippocampus of mice infected with *N. caninum*. Level of GABA, a major inhibitory neurotransmitter, was decreased in the cortex of infected mice suggesting that elevated Glu levels might be due to lack of effects of GABA. Our previous study showed activation of microglia in the brain of mice infected with *N. caninum*^16^. Activated microglia induce excess Glu and causes excitotoxicity to neuronal cells via the N-methyl-D-aspartate (NMDA) receptor^35^,^36^. Because GABA levels showed no difference in the caudoputamen and hippocampus between infected and uninfected mice, our result supports elevated Glu might be a result of an immune reaction mediated via activated microglia.

Release of IFN-γ leads to tryptophan degradation via indoleamine-2,3-dioxygenase and elevated 5-HT^42^. In contrast, our data showed increased level of 5-HT in almost all regions examined while the expression level of IFN-γ was highly upregulated in those areas, suggesting a possibility that tryptophan levels were increased in the brains of mice infected with *N. caninum*. It has been suggested that increased tryptophan levels are associated with infection and are often accompanied by 5-hydroxyindole acetic acid, a major metabolite of 5-HT^37^. In fact, *T. gondii*-infected male mice showed increased serotonergic activity 3 weeks post infection^41^. Interestingly, IL-1 and lipopolysaccharide (LPS) administration increase concentration of tryptophan and 5-HT throughout the brain^37^. In addition, LPS administration increased levels of DA and 5-HT^43^. Therefore, *N. caninum* infection induced marked change of monoamine levels in wide brain areas via long-lasting immune reactions.

Since noradrenergic and serotonergic systems function to inhibit active behavior^37^, elevated brain serotonergic neurons by *N. caninum* infection may contribute to behavioral changes. Furthermore, DA levels in the caudoputamen were increased in mice infected with *N. caninum*, while that in the thalamus was decreased. Both the caudoputamen and thalamus are part of the basal ganglia circuit which control voluntary motor movements^44^. Thus, dysregulation of dopaminergic neurons in these regions may contribute to behavioral changes associated with *N. caninum* infection.

Although differences in changes of neurotransmitter levels among brain regions were observed, further research is required to clarify the relationship between neurotransmitter level and parasite load and severity of brain lesions. In this study there were no data concerning the correlation between severity behavioral deficits and the amount of neurotransmitter change because mice tested in the open field were different from mice prepared for HPLC and qPCR analysis. However, we additionally analyzed the correlation between behavioral changes and parasite load (supplemental Table 1). We found that the number of parasites in the thalamus had a negative correlation with the total distance travelled and average speed in the open field test. In addition, the number of parasites in the caudoputamen showed a negative correlation with average speed in the open field test. The thalamus and caudoputamen have a connection to the motor cortex^44^. Thus, these results suggested that parasites in these regions might contribute to behavioral changes in *N. caninum*-infected mice. Furthermore, the number of parasites in the cerebellum had a negative correlation with rearing counts in the open field test. Normal cerebellum processing is necessary for motor control^44^. Therefore, our results suggested that parasite load in the cerebellum affect the total number of rearing counts.

Hind limb paralysis and rigid hyperextension are a characteristic symptom of *N. caninum* infection in dogs^4^,^5^,^6^. *N. caninum*-infected mice show a similar type of neurological symptom^13^,^14^,^15^. However, the mechanism of neurological symptom is almost unknown. Gly levels are altered by *N. caninum* infection in several brain regions. In addition, levels of Gly were increased in the hindbrain including pons and oblongata, midbrain and cerebellum of the infected mice. Gly is the major inhibitory neurotransmitter in the hindbrain and the spinal cord^45^. Increased Gly level in cerebrospinal fluid caused severe hyperkinesia in humans^46^. Thus, these results implicate that increased Gly levels in the progressive stage of neosporosis might cause hind limb paralysis.

The expression of IEGs is induced by neuronal activity. c-Fos is a regulatory transcription factor and a known marker of neuronal activity. Arc is an effector protein, a key protein implicated in synaptic plasticity and synapse strength and hence learning and memory^47^. In the present study, expression levels of c-Fos and Arc were decreased in *N. caninum*-infected mice, suggesting *N. caninum* infection downregulated gene expression associated with neuronal cell function. Thus, *N. caninum* infection may impair learning and memory capacity of its host. Because we confirmed the upregulation of IFN-γ and TNF-α expression and alteration of neurotransmitter levels in the brain of *N. caninum*-infected mice, downregulation of c-Fos and Arc expression in the brain may be triggered by neurological pathology following *N. caninum* infection. In conclusion, our results suggest that chronic neuroinflammation following *N. caninum* infection may cause neurological dysfunction and neuronal cell death, resulting in neosporosis.

## Methods

### Ethics Statement

This study was performed in strict accordance with recommendations in the Guide for the Care and Use of Laboratory Animals of Ministry of Educations, Culture Sports, Science and Technology, Japan. The protocol was approved by the Committee on the Ethics of Animal Experiments of the Obihiro University of Agriculture and Veterinary Medicine (Permit number 25–59, 25–60, 25–62). Mice were decapitated without anesthesia for brain sampling, and all efforts were made to minimize animal suffering.

### Mice

Male C57BL/6 mice (8 weeks old) were purchased from Clea Japan (Tokyo, Japan). Mice were housed (four to six mice/cage) in 12-h light (8:00–20:00) in the animal faculty of the National Research Center for Protozoan Diseases at Obihiro University of Agriculture and Veterinary Medicine, Obihiro, Japan. All mice were treated using the guiding principles for the care and use of research animals endorsed by the Obihiro University of Agriculture and Veterinary Medicine, Obihiro, Japan. All animal experiments began after 1 week of habituation.

### Preparation of *N. caninum* tachyzoites and parasite infection of mice

*N. caninum* (strain Nc-1) parasites were maintained in monkey kidney adherent epithelial cells (Vero cells) cultured in Eagle’s minimum essential medium (Sigma, St. Louis, MO, USA) containing 8% heat-inactivated fetal bovine serum (FBS). For the purification of the tachyzoites, the parasites and host-cell debris were washed in cold phosphate-buffered saline (PBS), and the final pellet was resuspended in cold PBS and passed through a 27-gauge needle and a 5.0-μm pore filter (Millipore, Bedford, MA, USA). *N. caninum* were intraperitoneally inoculated (1 × 10^5^ tachyzoites) into mice (9 weeks old). To check clinical condition, daily body weight measurements were taken for 30 days after infection.

### Open field test

To investigate activity and exploratory behaviour of *N. caninum*-infected mice, mice were tested one at a time. Exploration in open field, a circular area with a diameter of 50 cm (Muromachi, Tokyo, Japan), was recorded for 5 min using a video tracking system (Comp Act VAS ver. 3.0×, Muromachi). Total travelled distance, average speed, and rearing counts were measured. Behavioural experiments were performed at 30 dpi, and commenced at 7:00 am, under 300 lux light intensity.

### Brain sampling

For histopathological analysis, five infected mice were sacrificed at 45 dpi and their brains were rapidly removed. The brains were perfused with 4% paraformaldehyde solution. For quantitative PCR of the parasites, two uninfected mice and twelve infected mice were sacrificed at 54 dpi. Their brains were divided into eight different areas: cortex, hippocampus, caudoputamen, amygdala, thalamus, hypothalamus, midbrain, and cerebellum. The samples to be used for DNA extraction and quantitative PCR of parasites were stored at −20 °C until analysis. For quantitative reverse-transcription-PCR (qRT-PCR) and high-performance liquid chromatography (HPLC) analysis, six uninfected mice and six infected mice were sacrificed at 40 dpi. The half brains were divided into five regions: cortex, hippocampus, caudoputamen, amygdala and thalamus, and were then frozen at −80 °C until use. The sample from the right and left brain was used for qRT-PCR and HPLC, respectively. To measure Gly levels in the hindbrain regions, eight uninfected mice and twelve infected mice were sacrificed at 54 dpi. The brain samples were divided into three hindbrain regions, cerebellum, midbrain, and medulla oblongata plus pons, and were then frozen at −80 °C until analysis.

### Histopathological analysis

After fixation with 4% paraformaldehyde solution, brain samples were cut coronally, embedded in paraffin wax, sectioned at 4 μm, and then stained with hematoxylin and eosin. Pathological lesion severity was scored by the following scheme: 0, no lesion; 1, slight lesion; 2, mild lesion; 3, moderate lesion; and 4, severe lesion. An example of the scoring is shown in Fig. 2(a). Total scores (from 0 to 4) for four types of lesions (meningitis, perivascular cuffs, inflammatory cells including glial cells, macrophages and lymphocyte infiltration, and necrosis) in each region by a severe degree were determined. Pathological scores were calculated by adding each score for different lesions, with total scores ranging from 0 to 16. The total score for each region was used for data analysis.

### DNA extraction and quantitative PCR for the detection of *N. caninum* DNA

DNA was isolated from each brain region, and parasite counts were analysed by real-time PCR using the Nc5 gene, as described previously^48^. The primers complementary to the Nc5 gene of *N. caninum*: the forward primer spanning nucleotides 248–257 (5′-ACT GGA GGC ACG CTG AAC AC-3′) and the reverse primer spanning nucleotides 303–323 (5′-AAC AAT GCT TCG CAA GAG GAA-3′). PCR was performed using an ABI prism 7900HT sequence detection system (Applied Biosystems, Foster City, CA, USA), and amplification monitored using the SYBR Green method (Applied Biosystems). A standard curve was constructed with tenfold serial dilutions of *N. caninum* DNA extracted from 1 × 10^5^ parasites. The curve ranged from 10,000 to 0.01 parasites. Parasite number was calculated by plotting Ct values on the standard curve.

### qRT-PCR

Total RNA was extracted from mouse brain using TRI reagent (Sigma). First-strand cDNA was synthesised from 0.4 μg total RNA by SuperScript^®^ First-Strand Synthesis System for RT-PCR (Invitrogen, Mount Waverley, Australia). Real-time PCR was performed according to Applied Biosystems (Applied Biosystems), and amplification monitored using SYBR Green (Applied Biosystems). IEG primers have been described previously^49^: Arc, 5′-GGA GGG AGG TCT ACC GTC-3′ and 5′-CCC CCA CAC CTA CAG AGA CA-3′; c-fos, 5′- CGA AGG GAA CGG AAT AAG-3′ and 5′-CTC TGG GAA GCC AAG GTC-3′. IFN-γ, 5′-GAG GAA CTG GCA AAA GGA TG-3′ and 5′-TGA GCT CAT TGA ATG CTT GG-3′. TNF-α, 5′-GGC AGG TCT ACT TTG GAG TCA TTG C -3′ and 5′-ACA TTC GAG GCT CCA GTG AA -3′. The housekeeping gene, glyceraldehyde 3-phosphate dehydrogenase (GAPDH) mRNA was amplified in parallel (5′-TGT GTC CGT GGA TCT GA-3′ and 5′-CCT GCT TCA CCA CCT TCT TGA T-3′) and used as an internal standard. The optimal reference gene was selected based on the Cotton EST database (http://www.leonxie.com). Expression levels of each gene relative to GAPDH were calculated using the 2^ΔCt^ method (User Bulletin no. 2; Perkin-Elmer, Boston, MA, USA). Fold expression levels among brain regions were calculated relative to expression levels of the corresponding gene in the hippocampus of uninfected mice.

### HPLC

Each brain sample was homogenised using a bio-masher (Funakoshi, Tokyo, Japan), and then 300 μL/10 mg tissue of 0.2 M perchloric acid (containing 100 μM EDTA-2Na) was added. Both isoproterenol HCL (monoamine internal standard) and homoserine (amino acid internal standard) (both Sigma) were added. Homogenates were placed on ice for 30 min and then centrifuged at 20,000 × *g* for 15 min at 0 °C. Supernatants were mixed with 1 M sodium acetate to adjust the pH to 3.0, and filtered by Ultra free MC (Millipore). Final products were injected into the HPLC system HTEC-500 (electrochemical detector; Eicom, Kyoto, Japan), equipped with a SC-5ODS column for monoamines or a SA-5ODS column for amino acids. Chromatographs were analysed using PowerChrom software version 2.5 (eDAQ Pty Ltd., Densitone East, Australia).

### Statistical analysis

Statistical analysis was performed using GraphPad Prism 6 (GraphPad Software, San Diego, CA, USA). Statistical differences between two groups were analysed using two-tailed unpaired *t* tests. With three groups or more, statistical differences were determined using one-way ANOVA followed by Tukey’s multiple comparison test. *P* values < 0.05 represent statistical differences.

## Additional Information

**How to cite this article**: Ihara, F. *et al*. Changes in neurotransmitter levels and expression of immediate early genes in brain of mice infected with *Neospora caninum*. *Sci. Rep*. **6**, 23052; doi: 10.1038/srep23052 (2016).

## Supplementary Material

Supplementary Information
