# Supplementary material for: Changes in neurotransmitter levels and expression of immediate early genes in brain of mice infected with Neospora caninum
Source: Sci Rep. 2016 Mar 14;6:23052. doi: 10.1038/srep23052 (PMC4789785; doi:10.1038/srep23052)
Supplement: Supplementary Information [file srep23052-s1.pdf]

Changes in neurotransmitter levels and expression of immediate early genes in brain of mice infected with *Neospora caninum*

Fumiaki Ihara[1], Maki Nishimura[1], Yoshikage Muroi[2], Hidefumi Furuoka[2], Naoaki Yokoyama[1], Yoshifumi Nishikawa[1,*]

[1] National Research Center for Protozoan Diseases, Obihiro University of Agriculture and Veterinary Medicine, Inada-cho, Obihiro, Hokkaido 080-8555, Japan.

[2] Department of Basic Veterinary Medicine, Obihiro University of Agriculture and Veterinary Medicine, Inada-cho, Obihiro, Hokkaido 080-8555, Japan.

[*]Corresponding author

Yoshifumi Nishikawa

Tel.: +81-155-49-5642

Fax: +81-155-49-5643.

Email: nisikawa@obihiro.ac.jp

Table S1. Pearson's correlation coefficients for the behavior in the open field test and parasite load in different brain regions.

	Total distace travelled (r)	Average speed (r)	Rearing (r)
Cortex	-0.299	-0.303	-0.337
Caudoputamen	-0.283	-0.604*	-0.518
Hippocampus	-0.229	-0.384	-0.001
Thalamus	-0.579*	-0.658*	-0.360
Amygdala	0.008	-0.293	-0.491
Hypothalamus	-0.167	-0.432	-0.277
Midbrain	-0.129	-0.424	-0.565
Cerebellum	-0.092	-0.381	-0.629*

The correlation coefficients for the behavior in the open field test and the parasite load in different brain regions were calculated using the Pearson correlation coefficient, two-tailed t tests (*$p < 0.05$). After the open field test, some mice were used for the correlation analysis. The strength of the linear association between pairs of variables can be determined as follows using the Pearson correlation coefficient: $|r| = 0.70$, strong correlation; $0.5 < |r| < 0.7$, moderately strong correlation; and $|r| = 0.3–0.5$, weak to moderate correlation. *N. caninum*-infected mice, n = 12.
